# Structure and Topology Prediction of Phage Adhesion Devices Using AlphaFold2: The Case of Two *Oenococcus oeni* Phages

**DOI:** 10.3390/microorganisms9102151

**Published:** 2021-10-14

**Authors:** Adeline Goulet, Christian Cambillau

**Affiliations:** 1 Architecture et Fonction des Macromolécules Biologiques, Centre National de la Recherche Scientifique (CNRS), Campus de Luminy, Case 932, CEDEX 09, 13288 Marseille, France; christian.cambillau@univ-amu.fr; 2 Architecture et Fonction des Macromolécules Biologiques, Aix-Marseille Université, Campus de Luminy, Case 932, CEDEX 09, 13288 Marseille, France

**Keywords:** AlphaFold2, *Oenococcus oeni*, *Siphoviridae*, bacteriophages, protein structure, protein modelling

## Abstract

Lactic acid bacteria (LAB) are important microorganisms in food fermentation. In the food industry, bacteriophages (phages or bacterial viruses) may cause the disruption of LAB-dependent processes with product inconsistencies and economic losses. LAB phages use diverse adhesion devices to infect their host, yet the overall picture of host-binding mechanisms remains incomplete. Here, we aimed to determine the structure and topology of the adhesion devices of two lytic siphophages, OE33PA and Vinitor162, infecting the wine bacteria *Oenococcus oeni*. These phages possess adhesion devices with a distinct composition and morphology and likely use different infection mechanisms. We primarily used AlphaFold2, an algorithm that can predict protein structure with unprecedented accuracy, to obtain a 3D model of the adhesion devices’ components. Using our prior knowledge of the architecture of the LAB phage host-binding machineries, we also reconstituted the topology of OE33PA and Vinitor162 adhesion devices. While OE33PA exhibits original structures in the assembly of its bulky adhesion device, Vinitor162 harbors several carbohydrate-binding modules throughout its long and extended adhesion device. Overall, these results highlight the ability of AlphaFold2 to predict protein structures and illustrate its great potential in the study of phage structures and host-binding mechanisms.

## 1. Introduction

Lactic acid bacteria (LAB)-infecting bacteriophages (phages or bacterial viruses) use diverse host-binding mechanisms, yet the overall picture of the interactions between LAB phages and their host remains incomplete. Unraveling the molecular details of phage–LAB interactions is essential not only for decoding phage biology but also for industrial and public health purposes, since LAB are important microorganisms in food fermentation and in the human gut microbiota. In the food industry, phages may disrupt LAB-dependent processes, causing serious concomitant economic losses. This is the case in dairy plants, where phages infecting the LAB species *Lactococcus lactis* and *Streptococcus thermophilus* are problematic and have therefore experienced extensive scientific scrutiny [1,2,3]. In contrast, there is a significant knowledge gap regarding the interactions between plant-associated LAB and their phages, even though they may also have a significant impact on fermentation processes and the quality of final products. This is true for fermented beverages, as exemplified by the emblematic and economically important field of winemaking, which relies heavily on the LAB species *Oenococcus oeni.* Recently, we have shown that representative phages that infect *O. oeni* possess host-binding devices with distinct compositions and morphologies [4,5] that differ from those of lactococcal and streptococcal phages. Moreover, wine polyphenolic compounds (PCs), which are abundant in the *O. oeni* ecological niche, can interfere with the phage infection process, as they may mimic some components of host cell wall polysaccharides (CWPS) [5]. Interestingly, some PCs prevent OE33PA, but not Vinitor162, from infecting *O. oeni*, indicating that these phages use different host-binding mechanisms.

Siphophages binding to host CWPS possess adhesion devices with some conserved features [1]. These phages are composed of four structural modules: (1) the capsid in which the DNA is packaged, (2) the connector that connects the capsid to the tail and controls DNA release, (3) the tail, consisting of stacked hexamers of the major tail protein (MTP), which is followed at its distal end by (4) the adhesion device. To date, it has been observed that the last MTP ring is attached to one (or two) distal tail (Dit) protein hexamer(s), followed by a trimer of Tail-associated lysozyme (Tal). The minimum size of Dits is about 120–140 residues, which corresponds to the size of a single belt domain [6]. However, most of them also possess an additional galectin [6,7] or OB-fold domain [8]. On top of these non-functional domains, extra carbohydrate-binding modules (CBM) can be found in the so-called evolved Dits that may contribute to CWPS binding [9,10]. Tals can be short, with a minimal length of 330 to 380 residues [11], or very long, with as many as 2000 residues [2]. They often harbor several CBMs along their extension and at their extremity [4,12,13]. This assembly can be completed by other proteins co-involved in CWPS binding, including one or several receptor-binding proteins (RBP) [2,7,14]. It is worth noting, in most cases, that the length of adhesion device proteins is between that of the long tape measure protein (TMP) and that of the holin/lysin cassette [1]. In general, but not always, these appear in the order *tmp, dit, tal, rbp*, and genes encoding ancillary proteins, making their initial identification fairly easy.

While X-ray diffraction of crystallized samples has been the key method used to determine phage protein 3D structures for a long time, this method has recently been challenged by cryo-electron microscopy (cryoEM) and single-particle analysis. The whole phage or large subcomplexes of it were the target of cryoEM studies [12,15,16]. However, in the last few weeks, the structure prediction software AlphaFold2 developed by the DeepMind company has become publicly available and may change the landscape of phage structural studies [17,18,19]. AlphaFold2 has revealed top capacity structure prediction in the challenging 14th Critical Assessment of protein Structure Prediction (CASP14) [20], greatly outperforming other methods.

In this report, we explored the capacity of AlphaFold2 to predict the structure and topology of different adhesion devices of the *O. oeni*-infecting phages OE33PA and Vinitor162. Our results demonstrate that AlphaFold2 complements HHpred analyses [21], since it offers the possibility of generating high-confidence 3D structures and, hence, to address host-binding mechanisms.

## 2. Materials and Methods 

### 2.1. Protein Structure Predictions

A user-friendly interface for accessing AlphaFold2 has recently been made available through notebooks. We used the ColabFold notebook, whose structure prediction is powered by AlphaFold2 combined with a fast, multiple sequence alignment generation stage using MMseqs2 [22,23]. Furthermore, this ColabFold allows the modeling of homo- or hetero-complexes. Due to memory limitations, we split long protein sequences (e.g., Vinitor162 Tal, 2170 residues long) into smaller stretches of 500–800 residues. Once individual domains were identified, we performed additional predictions using each domain sequence as an input. Due to phage structural proteins often being homomultimers, we systematically predicted hexameric or trimeric assemblies when possible within memory limitations. In parallel, homo-multimerization was also predicted with SymmDock [24]. The final predicted domain structures were submitted to the Dali server to identify the closest structural homologs in the PDB [25]. Lastly, the sequences submitted to ColabFold were also submitted to HHpred [21] in order to compare the domain boundaries and folds with those predicted by AlphaFold2. Views of the domain 3D structures were prepared with ChimeraX [26].

### 2.2. Adhesion Device Topology Predictions

Symmetric assemblies of adhesion device components were predicted either by AlphaFold2 (e.g., the Vinitor162 Dit hexamer and the OE33PA RBP trimer) or by SymmDock (Vinitor162 Tal *β*-prisms and *β*-helices). We used the *Coot* option “SSM Superpose” to superimpose individual domains onto the corresponding ones of the lactococcal phage p2 adhesion device [7,27]. In the OE33PA adhesion device, we positioned the CBM and its flanking *β*-domains without linkers connecting them to each other and to the belt and galectin domains. In the Vinitor162 adhesion device, the CBM_1 orientation in the Tal structural domain was that originating from AlphaFold2, and the trimeric assemblies of CBM_2-5 were provided by SymmDock. The assembly of the Tal C-terminal end was produced with SymmDock for the *β*-prisms and *β*-helices domains, as well as with AlphaFold2 for the chaperone domain. These modules were placed according to their sequence order along the Tal extension, as viewed by negative staining electron microscopy (nsEM) [4]. The figures of the OE33PA and Vinitor162 adhesion device topology predictions were prepared with ChimeraX [26].

## 3. Results

**Figure 1:**
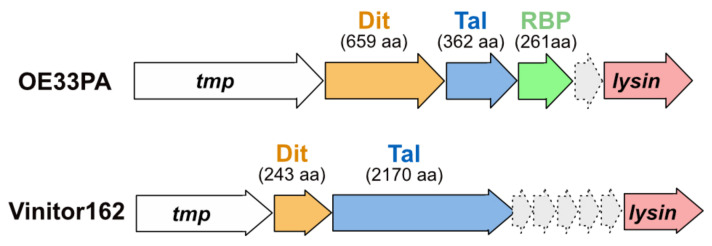
Schematic representation and assignment of adhesion device-encoding genome regions. Genes encoding adhesion device components are located between *tmp* and *lysin*. Orange arrows represent open-reading frames (ORFs) encoding Dits, blue arrows represent ORFs encoding Tals, and the green arrow represents the ORF encoding the OE33PA RBP. Light grey arrows represent the ORFs encoding hypothetical proteins. The number of protein residues involved is indicated (aa: amino acids).

The oenococcal phages OE33PA and Vinitor162 possess distinct adhesion devices that differ in many aspects from those of lactococcal and streptococcal phages. In previous studies, we have shown that the OE33PA adhesion device has three components: Dit, Tal, and RBP. Meanwhile, only two components, Dit and Tal, make up that of Vinitor162 [4] (Figure 1).

Here, we used the state-of-the-art machine learning method AlphaFold2 in order to obtain high-confidence structure and topology predictions of these adhesion devices and thereby address the structure–function relationships of these macromolecular machineries. We compared these predictions to HHpred analyses and used them to propose topological models of the OE33PA and Vinitor162 adhesion devices based on prior knowledge of the architecture of phage tail tips and host-binding machineries (Figure S1).

### 3.1. Phage OE33PA

We submitted the sequences of Dit, Tal, and RBP to AlphaFold2 for structure prediction.

#### 3.1.1. An Evolved Dit

The Dit of OE33PA is 659 residues long, much longer than the so-called classical Dits, such as those of *L. lactis* phage p2 (298 residues) or *B. subtilis* phage SPP1 (253 residues) [1]. First, we submitted the full-length Dit sequence to AlphaFold2. This returned a 3D model encompassing five domains (Figure S2). Based on this model, we split the Dit sequence into five parts and submitted each of them to AlphaFold2. With the aim of obtaining more accurate domain boundaries and structure predictions, we analyzed sequence 1 (1–173), sequence 2 (180–272), sequence 3 (290–445), sequence 4 (455–517), and sequence 5 (520–659).

**Figure 2:**
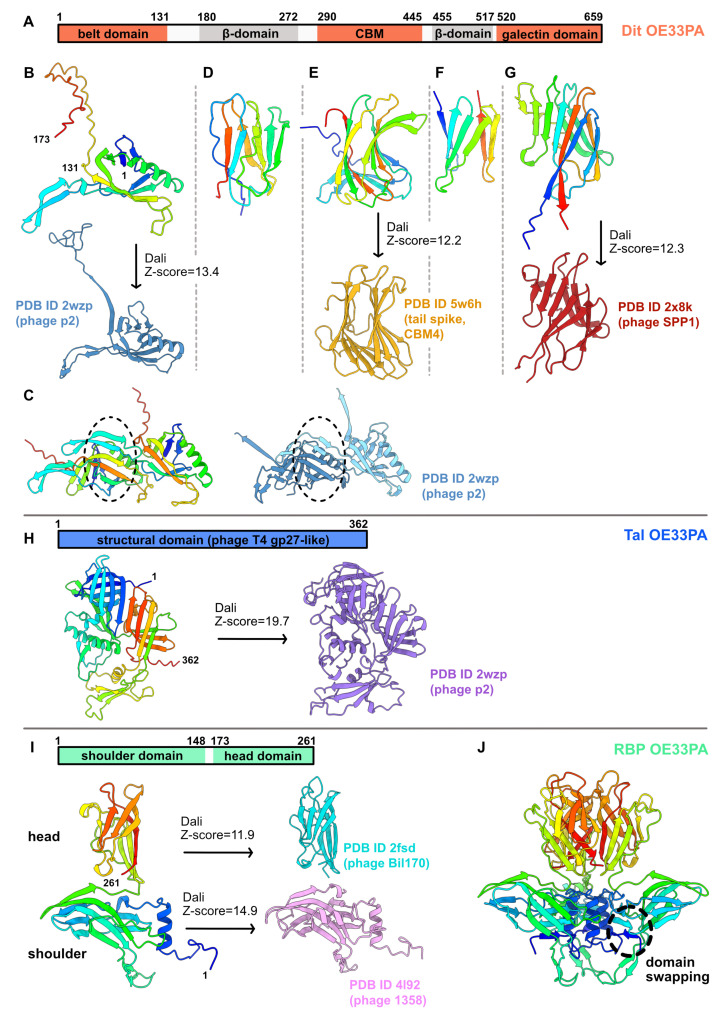
AlphaFold2 structure predictions for OE33PA Dit, Tal, and RBP. (**A**–**G**) Dit of OE33PA. (**A**) Dit domain organization. Domains with structural homologs are shown in salmon, while domains with unknown folds are shown in grey. The domain boundaries predicted by AlphaFold2 are indicated. (**B**) Ribbon representations of the predicted belt and putative ‘arm’ structure (rainbow) and of the phage p2 Dit belt–arm domain (steel blue). (**C**) Comparison of the Dit dimeric assembly predicted by AlphaFold2 (rainbow) and a p2 Dit dimeric assembly determined by X-ray crystallography (steel bleu). Dotted ovals highlight intermolecular contacts. (**D**) Ribbon representation of the predicted *β*-domain (rainbow). This structure did not return significant hits with Dali. (**E**) Ribbon representations of the predicted CBM (rainbow) and the C-terminal domain of a phage tail spike (gold). (**F**) Ribbon representation of the predicted *β*-domain (rainbow). This structure did not return significant hits with Dali. (**G**) Ribbon representations of the predicted galectin structure (rainbow) and the phage SPP1 Dit galectin domain (red). (**H**) (Top) Tal domain organization. (Bottom) Ribbon representations of the predicted Tal structure (rainbow) and the phage p2 Tal structural domain (purple). (**I**) (Top) RBP domain organization. (Bottom) Ribbon representations of the predicted RBP structure (rainbow), the phage Bil170 RBP head domain (pink), and the phage 1358 RBP shoulder domain (light blue). (**J**) Ribbon representations of the AlphaFold2 RBP trimeric assembly. The dotted oval highlights shoulder domain swapping.

Sequence 1 returned the typical N-terminal belt domain (1–131) of Dit proteins with a high confidence score (Figure 2A,B and Figure S3). Moreover, an elongated structure (131–173) reminiscent of the ‘arm’ in the Dit of phage p2, which holds a trimer of RBPs [7], may extend the belt domain (Figure 2B). This 3D model returned significant hits when using the Dali server [25] with the Dit of phages p2 and SPP1 (Table S1). Moreover, AlphaFold2 also predicted a reliable intermolecular assembly with the formation of a protein–protein interface similar to that observed in the Dit hexameric rings of phages T5, SPP1, p2, TP901-1, and 80α [6,7,8,12,14] and of a gene transfer agent [16] (Figure 2C). Interestingly, sequence 5 folds into the typical C-terminal galectin domain (526–659) of Dit proteins (Figure 2G and Figure S3, Table S1). Therefore, these predictions highlight that the evolved Dit of OE33PA contains several domains inserted between its N-terminal belt domain and C-terminal galectin domain.

The structures of sequences 2, 3, and 4 were predicted with poor confidence scores because of their low sequence coverages (Figure S3). However, the predicted *β*-sandwich domain for sequence 3 returned significant hits with CBMs—with which it shares a typical “U-shape”—using Dali (Figure 2E, Table S1). It is noteworthy that the top hit corresponds to the C-terminal domain of a RBP from the coliphage CBA120 [28]. This indicates that the evolved Dit of OE33PA contains one CBM, likely CBM4 according to the CAZy nomenclature [29], which may be involved in the recognition of host CWPS. The domain connecting the CBM to the belt domain (corresponding to sequence 2) returned a hit with a fibronectin-binding domain (Table S1), while the domain connecting the CBM to the galectin domain (sequence 3) did not return significant structural homologs using Dali. Therefore, these predicted *β*-sandwiches may be structural domains that contribute to the overall architecture of the protein. Lastly, the HHpred analyses of the OE33PA Dit appeared overall in agreement with the AlphaFold2 domain boundaries and structure predictions (Table S1).

#### 3.1.2. A Short Tal

The Tal of OE33PA is 362 residues long, which makes it a short Tal [1]. AlphaFold2 returned a reliable structure prediction for the full-length sequence that closely matches the Tal of phage p2 (Figure 2H, Figure S3, Table S1). Therefore, the Tal of OE33PA corresponds to the structural domain of Tal proteins, which is related to the baseplate hub protein gp27 from myophage T4 [30]. Surprisingly, the HHpred analysis reported significant hits (probabilities >99%) with short Tal from various phages but not with the Tal from phage p2 (Table S1).

#### 3.1.3. A Chimeric RBP

The RBP of OE33PA is 261 residues long, which is in the range of the RBPs from other LAB phages [1]. We submitted the full-length sequence to AlphaFold2, producing a typical RBP structure encompassing the shoulder and the head domains, with the latter being involved in host binding (Figure 2I, Table S1). Interestingly, the shoulder domain (1–148) is similar to that of the lactococcal phage 1358 RBP [31], while the head domain (157–261) is similar to that of lactococcal phages (TP901-1, Bil170) and *Listeria* phage PSA RBPs [14,32,33].

These domains are connected by a short linker, as also observed for the phage 1358 RBP. AlphaFold2 also returned a trimeric assembly compatible with the canonical oligomerization state of phage RBPs (Figure 2J). Moreover, domain swapping between shoulder domains, as observed in the crystal structure of the phage 1358 RBP, was properly predicted by AlphaFold2 (Figure 2J). Therefore, OE33PA RBP presents a chimeric structure sharing domains with RBPs from different Gram-positive infecting phages. It is noteworthy that this chimeric organization was also identified by HHPred with a high probability (>98%), even though the top hit corresponded to the full-length phage p2 RBP, in which the neck domain is an elongated *β*-helix [7] (Table S1).

#### 3.1.4. Adhesion Device Topology: A Bulky, Star-like Assembly

**Figure 3:**
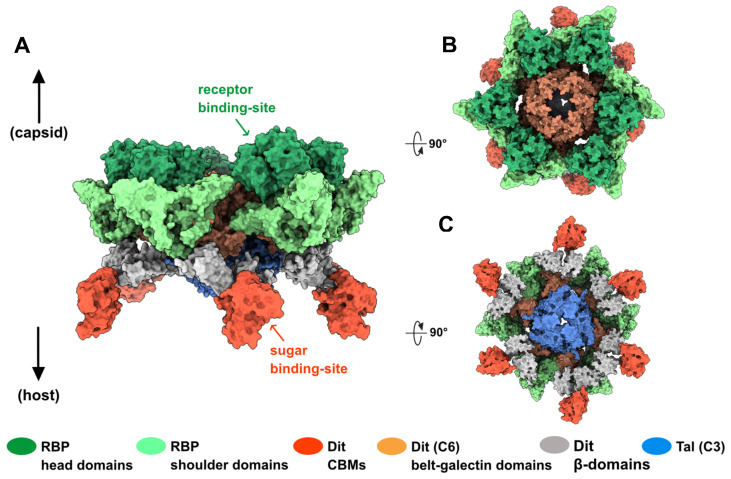
Topological model of the OE33PA adhesion device. (**A**–**C**). Orthogonal views of the adhesion device (**A**): side view; (**B**): top view; (**C**): bottom view). This model was generated from the crystal structure of the phage p2 adhesion device in its resting state with the RBP head domains pointing towards the capsid (PDB ID 2wzp). Connections between CBMs (red) and their flanking *β*-domains (grey) are not represented. The color code is indicated.

We used the AlphaFold2 structure predictions of Dit, Tal, and RBP to propose a topological model of the OE33PA adhesion device (Figure S1). Since these proteins are similar to those found in lactococcal phages with ‘activable’ adhesion devices [7,34], we based our model on the phage p2 adhesion device. We used the crystal structure of the p2 adhesion device in its resting state in order to highlight the different roles likely played by RBPs and CBMs upon host recognition and binding (Figure 3). First, we split the OE33PA Tal monomer into two regions (region 1: residues 1–213 and 214–362; region 2: residues 214–291) and superimposed each of them on the p2 Tal trimer, thereby assembling a closed OE33PA Tal trimer. Indeed, movements in region 2 were observed upon Tal opening [7]. Additionally, we superimposed OE33PA RBP trimers onto the six p2 RBP trimers, and the Dit belt and galectin domains onto the p2 Dit hexameric ring. Then, we positioned six CBMs and their flanking *β*-domains close to the galectin domains, at the periphery of the Dit–Tal–RBP assembly. In this configuration, CBMs, with their sugar-binding sites pointing outwards the adhesion device, could interact with the host CWPS more easily than the RBPs could (Figure 3). Moreover, interactions between CBMs and their receptors might trigger Dit conformational changes and, consequently, the reorientation of RBPs towards the host cell wall for irreversible host binding.

### 3.2. Phage Vinitor162

Vinitor162 has only two putative proteins building its adhesion device: a Dit and a long and extended Tal [4].

#### 3.2.1. A Classical Dit

**Figure 4:**
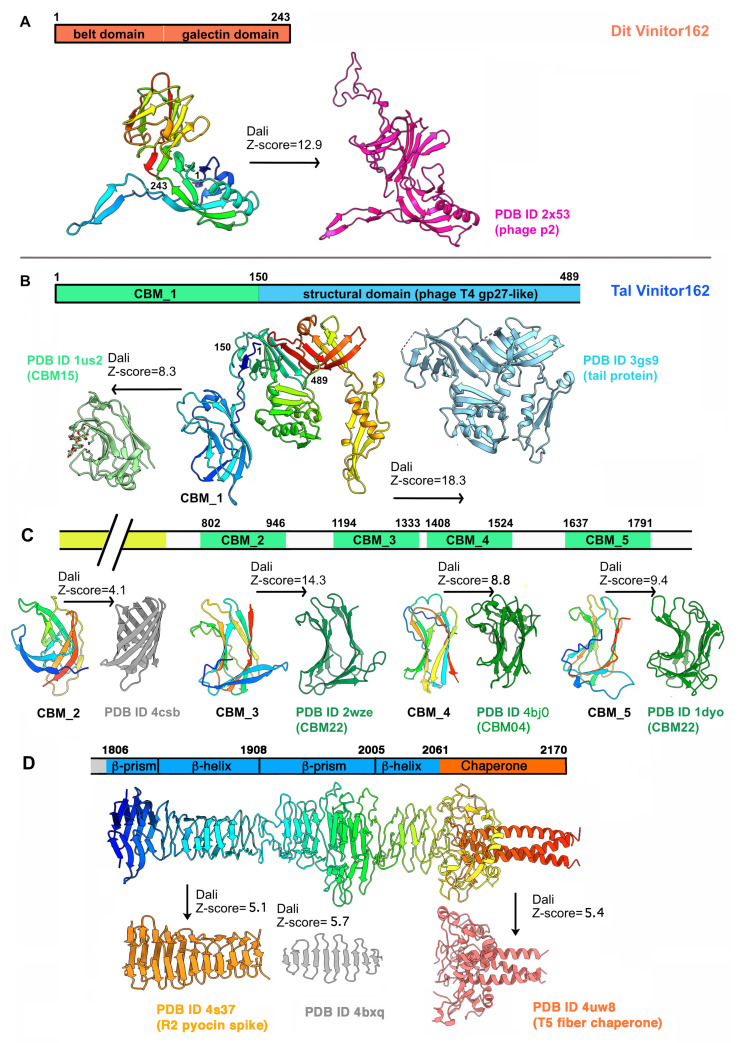
AlphaFold2 structure predictions of Vinitor162 Dit and Tal. (**A**) Dit domain organization (top). Ribbon representations of the predicted belt and galectin domains (rainbow) and the phage p2 Dit belt–arm and galectin domains (pink). (**B**) Tal domain organization in the N-terminal region (top). Ribbon representations of the Tal and CBM_1 predicted structure s(rainbow) and their Dali hits, the listerial phage EGD-e Tal structural domain (light blue), and a CAZy CBM15 (light green), respectively. (**C**) Tal domain organization in the middle of the protein (top). Ribbon representations of the predicted CBM_2 to CBM_5 (rainbow) and their Dali hits. Besides CBM_2, the three other CBMs correspond to CAZy CBM22, CBM4, and CBM22. (**D**) Tal domain organization in the C-terminal region (top). Ribbon representations of the predicted Tal C-terminal domain (rainbow) compared to Dali hits, a R2 pyocin spike (orange), and a T5 fiber chaperone (pink) at each extremity.

The Dit of Vinitor162 is 243 residues long, making it a classical Dit [1]. AlphaFold2 prediction returned a canonical Dit structure with belt and galectin domains, devoid of any insertions (Figure 4A and Figure S4). It also predicted an hexameric assembly resembling that of the Dits of known structures [7,8,14,16]. When submitted to Dali, a long list of hits with high Z-scores was returned, all related to phage Dits. The best hit aligned with the belt and galectin domains of the Dit of lactococcal phage p2 (Table S1). However, while an ‘arm’ is present in the Dit of phage p2 to anchor a trimer of RBP, it is absent in the Dit of SPP1 and Vinitor162, which are both devoid of RBP per se. Moreover, HHpred analysis returned the Dit of phage SPP1 as a hit with a 100% probability covering the entire protein (Table S1). 

#### 3.2.2. A Long and Multi-Domain Tal

The Tal of Vinitor162 is 2170 residues long. Therefore, we split its sequence into four parts for AlphaFold2 analysis. The first part (residues 1–525) returned a model composed of two domains (residues 1–480) and a terminal α-helix (Figure 4B). When submitted to Dali, the 1–150 segment was identified as a CBM (hereafter named CBM_1), i.e., CBM15, according to the CAZy nomenclature [29], while residues 151–480 were ascribed to the structural domain of a short Tal protein from *Listeria monocytogenes* EGD-e (Figure 4B and Figure S4, Table S1). The second part (residues 500–1000) returned a long α-helical domain (490–801) followed by a globular *β*-stranded domain (802–946). The latter exhibits the same “U-shape” as CBM_1. However, it was not identified by Dali as a CBM but as a bacterial *β*-barrel protein (Figure 4C, Table S1), a result that could be due to a poor prediction (Figure S4) or to the presence of a new fold. The third part, including residues 900 to 1550, reported two juxtaposed CBMs named CBM_3 and CBM_4. In this model, CBM_3 (1194–1333) is linked to CBM_4 (1407–1525) by a compact linker placing them side by side. The Dali analysis of CBM_3 returned a CAZy CBM22 fold, while that of CBM_4 returned a hit with a CAZy CBM4 (Figure 4C, Table S1). The last part (1500–2170) generated feature-rich domains. First, a fifth CBM structure (CBM_5) was produced between residues 1637 and 1791. CBM_5 was also ascribed to CAZy CBM22 by Dali, as for CBM_3 (Figure 4D and Figure S4, Table S1). After a short linker, a series of structures characteristic of phage tail tips or tail fibers were predicted at the C-terminal end for residues 1806–2170. The stretch 1806–1908 features a *β*-prism domain followed by a longer *β*-helical domain (Figure 4D). This fold was identified by Dali as resembling the R2 pyocin membrane piercing spike (PDB ID 4s36; (Browning, C.B.; Leiman, P.G.; and Shneider, M.M., unpublished) (Table S1). However, AlphaFold2 was unable to assemble three monomers into an interlaced *β*-helical domain, as found in phages. Instead, we used SymmDock, a server for the prediction of complexes with Cn symmetry by geometry-based molecular docking [24], to generate this interlaced structure (Figure 4D). Similarly, the stretch 1908–2061 displays a large *β*-prism structure, followed by a short, poorly defined, interlaced *β*-helix that was assembled as a trimer by SymmDock (Table S1). Lastly, the C-terminal domain, encompassing residues 2062–2170, displays a domain rich in loops with few *β*-strands, terminated by a long α-helix, which was predicted as a trimeric assembly by AlphaFold2 (Figure 4D). When submitted to Dali, the C-terminal receptor-binding domain (RBD) of the coliphage T5 L-shaped tail fiber was retrieved [35] (Table S1). In particular, it is the uncleaved chaperone present in this T5 component that matches with the C-terminal domain of the Vinitor162 Tal. Finally, the whole-length analysis by HHpred reported hits only at the N-terminal and C-terminal ends. In contrast, when splitting the analysis in four stretches, more features appeared, as reported in Table S1.

#### 3.2.3. Adhesion Device Topology: An Elongated, CBM-Rich Assembly

**Figure 5:**
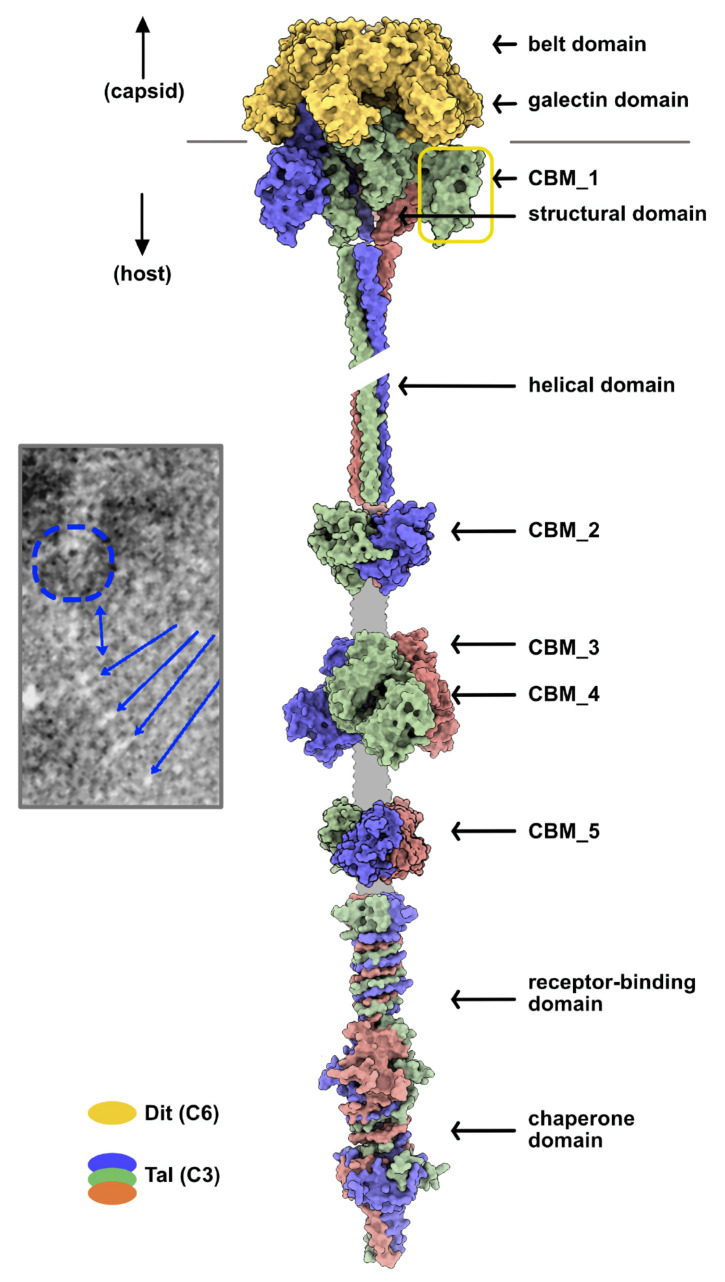
Topological model of the Vinitor162 adhesion device. This model was generated from the Dit hexamer and Tal trimer in the crystal structure of the phage p2 baseplate in its resting state. The trimerized CBMs were added to the sequence along the elongated structure. The color code is indicated, and the unpredicted linkers are shown in grey. Inset: nsEM image of Vinitor162 tail tip [4]. The putative location of the Tal structural domain is indicated by a dotted blue circle, the elongated helical domain is indicated by a blue double arrow, and CBM_2-5 and the C-terminal domain are indicated by blue arrows.

We produced a Vinitor162 adhesion device topological model in several different steps. First, six Dit monomers were assembled as a hexamer with *Coot*, using the crystal structure of the phage p2 adhesion device as a template [7]. Then, using the same template, we assembled a trimeric Tal N-terminal region including the Tal structural domain and CBM1 (residues 1–489). For the rest of the Tal, the folded segments, long helix, CBM_2, CBM_3-4, and CBM_5 were independently assembled as trimers using SymmDock. The C-terminal domains (residues 1806–2170) were assembled either with SymmDock (residues 1806–2061) or with AlphaFold2, and their trimeric structures were juxtaposed in sequence (Figure 5).

## 4. Discussion

Studying siphophage structures is difficult due to the flexible nature of their long tail. Therefore, a “divide-and-conquer” approach is often used to tackle this problem and determine the 3D structures of phage tail components, including multiprotein host-adhesion devices. In particular, X-ray crystallography and cryoEM have revealed atomic details of such assemblies, thereby providing the molecular basis of phage–host interactions [1,11,36]. In this context, AlphaFold2, which proved to be highly reliable for protein structure prediction [19], is a perfect tool for the analysis of multi-domain proteins, such as those forming the adhesion device of siphophages.

Host-adhesion devices are mosaic assemblies built around a common scaffold encompassing a Dit hexameric ring at the tail distal end and a Tal trimer attached to it. This Dit–Tal scaffold serves as a platform to adapt dedicated RBPs and ancillary proteins. The model lactococcal phage p2 comprises Dit, Tal, and RBPs [7], while other phages, including the lactococcal phages TP901-1 [14] and Tuc2009 [37] and the staphylococcal phage 80α [12], assemble more components. In particular, CBMs are commonly found in adhesion devices as well as in the capsid, neck passage structure, and tail tube for the preliminary, reversible saccharide binding involved in putative host scanning [10]. This composition diversity, which is directly reflected in host-binding mechanisms, is also found in *O. oeni*-infecting phages: the OE33PA host-adhesion device is composed of Dit, Tal, and RBP, while the Vinitor162 host-adhesion device consists only of the two core components. However, the nsEM imaging of Vinitor162 has revealed a highly flexible Tal extension showing several bulbs interpreted as CBMs, likely involved in host binding [4].

Overall, the OE33PA adhesion device shares similarities with those of well-known lactococcal phages. Notably, it appears as an ‘activable’ adhesion device, similar to that of lactococcal phages p2 and 1358 [7,34], and likely uses RBP and CBM to bind specifically to the host. Additionally, it is striking how a rather limited number of structural “bricks” can produce diverse and host-specific adhesion devices. This is clearly illustrated in the OE33PA RBP and evolved Dit. The RBP is formed by a shoulder domain found in the lactococcal phage 1358 [38] and a head domain found in the lactococcal phages of the *Skunavirus* genus (former 936 group) [7,33] and P335 group [14] or in listerial phages [32]. Therefore, RBP attachment to the OE33PA Dit–Tal core likely engages a Dit ‘arm’, as observed in p2 and 1358 adhesion devices [7,34,39]. Regarding the evolved Dit, it contains one CBM insertion, similar to many other phages of the *Skunavirus* genus [10]. It is noteworthy that the OE33PA Dit CBM is predicted to be a CBM4, like the ancillary protein BppA of the lactophage Tuc2009 (P335 group) involved in host cell binding [37]. However, the OE33PA Dit CBM is inserted between the belt and galectin domains and flanked by *β*-sandwich domains, while the CBMs identified in the evolved Dits of skunaviruses are inserted within the Dit ‘arm’ [10]. Like the dynamic Tuc2009 BppA located at the periphery of the adhesion device [37], the OE33PA CBM may point outwards of the Dit ring. Moreover, this CBM is connected to the core of the adhesion device via a long linker (Figure 2A), which likely makes it dynamic and facilitates its interaction with the host CWPS.

The Dit of Vinitor162 resembles that of phage p2, but without the ‘arm’ extension, and that of phage SPP1 that also lacks this extension. It does not harbor CBMs, as seen in OE33PA and many other skunaviruses. In contrast with the Tal of OE33PA, the Tal of Vinitor162 is very long, encompassing 2170 residues. Such long Tals are not rare and were previously identified in the P335 group of lactococcal phages and in the *Streptococcus thermophilus* phages pac and cos. The analysis of these long Tals with HHpred indicated that they may incorporate several CBMs [2,40]. AlphaFold2 predicted up to five different modules along the Tal extension and an RBD at the Tal tip, followed by a chaperone domain. Four of the five modules were identified unambiguously as CBMs by Dali, while CBM_2 was not, although it exhibits the classical U-shape of CBMs. The Tal tip resembles the C-terminal domain of the phage T5 L-shaped fiber involved in preliminary, reversible phage adhesion to the host [35]. The chaperone domain ensures the proper folding of the fiber and is afterwards cleaved by the *β*-helical domain.

The high number of adhesion modules in the Tal of Vinitor162 is remarkable, as it accounts for 15 CBMs (5 per Tal monomer) and 3 RBDs. However, this total of 18 adhesion modules is comparable to the number of RBPs in the skunavirus p2 (6 RBP trimers) and in OE33PA. In skunaviruses, the specificity of a few of these CBMs towards their hosts was determined by host cell-binding assays using fluorescently labelled CBMs. In each case, the ancillary CBMs exhibited the same specificity as that of the bona fide RBP [10,41]. It remains to be determined, however, whether the ancillary CBM of OE33PA exhibits the same host specificity as that of RBP. Similarly, it is not known whether all Vinitor162 CBMs are functional or whether they have the same host specificity as the RBD. Here, AlphaFold2 predicted CBM domain boundaries with a higher precision than HHpred, which is a prerequisite for the successful recombinant expression of these domains in *E. coli* and subsequent fluorescence host cell-binding assays.

## Data Availability

Not applicable.

## Supplementary Materials

The following are available online at https://www.mdpi.com/article/10.3390/microorganisms9102151/s1: Figure S1: Workflow for structure and topology predictions. Figure S2: AlphaFold2 structure prediction of the OE33PA full-length Dit. Figure S3: AlphaFold2 confidence measures for OE33PA Dit domains, Tal, and RBP. Figure S4: AlphaFold2 confidence measures for Vinitor162 Dit and Tal domains. Table S1: Comparison of domain predictions by HHpred and AlphaFold2.
