# Supplementary material for: Structure and Topology Prediction of Phage Adhesion Devices Using AlphaFold2: The Case of Two Oenococcus oeni Phages
Source: Microorganisms. 2021 Oct 14;9(10):2151. doi: 10.3390/microorganisms9102151 (PMC8540738; doi:10.3390/microorganisms9102151)
Supplement: Supplementary file 1 [file microorganisms-09-02151-s001.zip › microorganisms-1404737-supplementary.pdf]

Structure and topology prediction of phage adhesion devices using AlphaFold2: the case of two *Oenococcus oeni* phages

Adeline Goulet[*] and Christian Cambillau

***Correspondence: adeline.goulet@univ-amu.fr; Tel.: 0033.491825590**

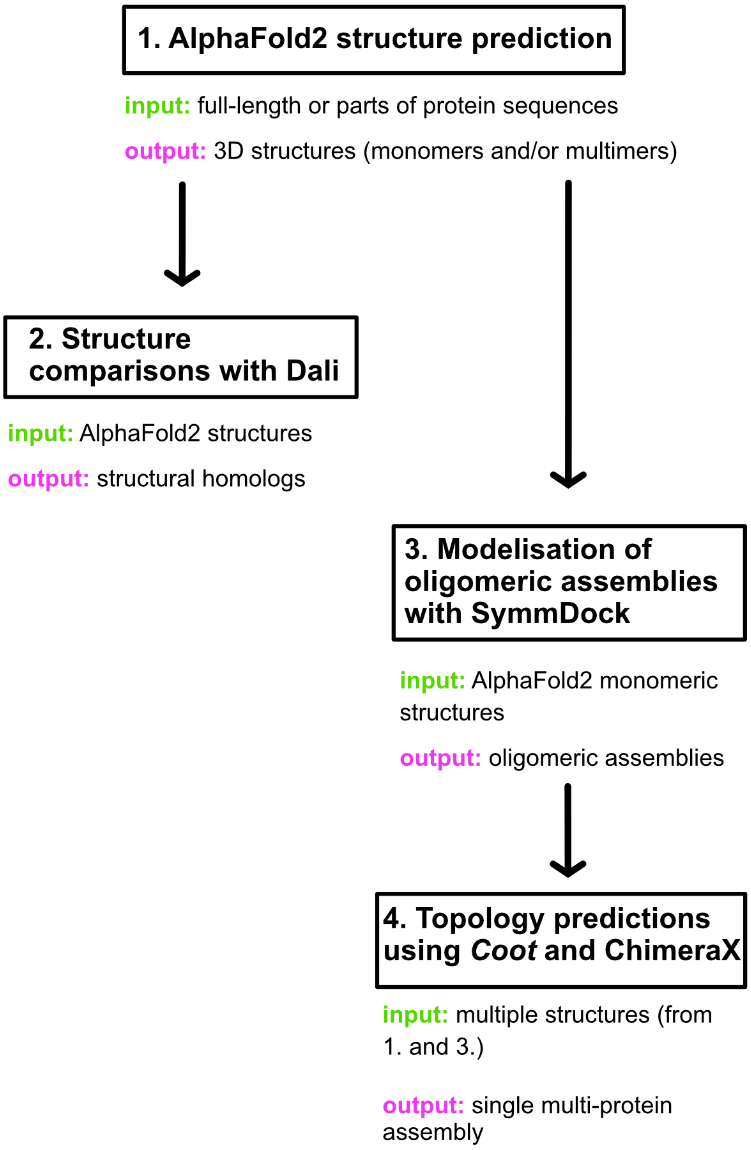

Figure S1. Workflow for structure and topology predictions. The different steps carried out for the structure and topology predictions are presented. The software used as well as their inputs and outputs are indicated.

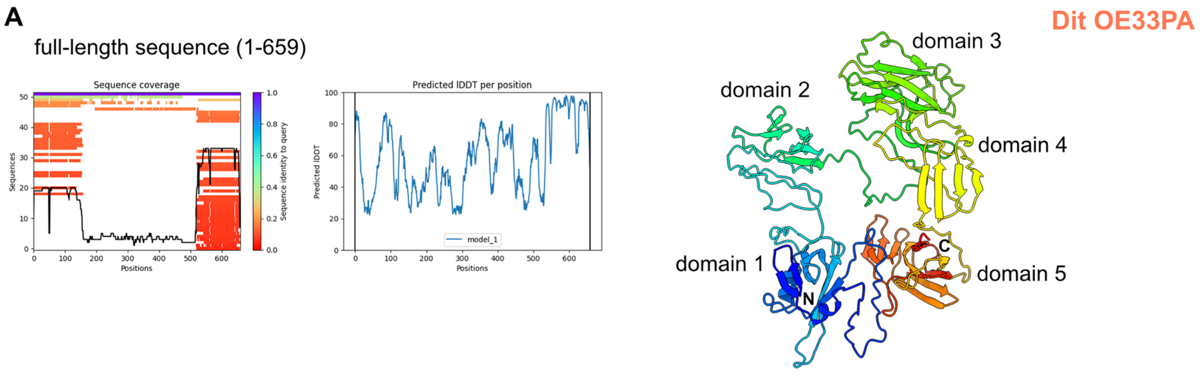
A
full-length sequence (1-659)
Sequence coverage
Sequences
Sequence identity to query
Positions
Predicted lDDT per position
Predicted lDDT
model_1
Positions
Dit OE33PA
domain 1
domain 2
domain 3
domain 4
domain 5
N
C

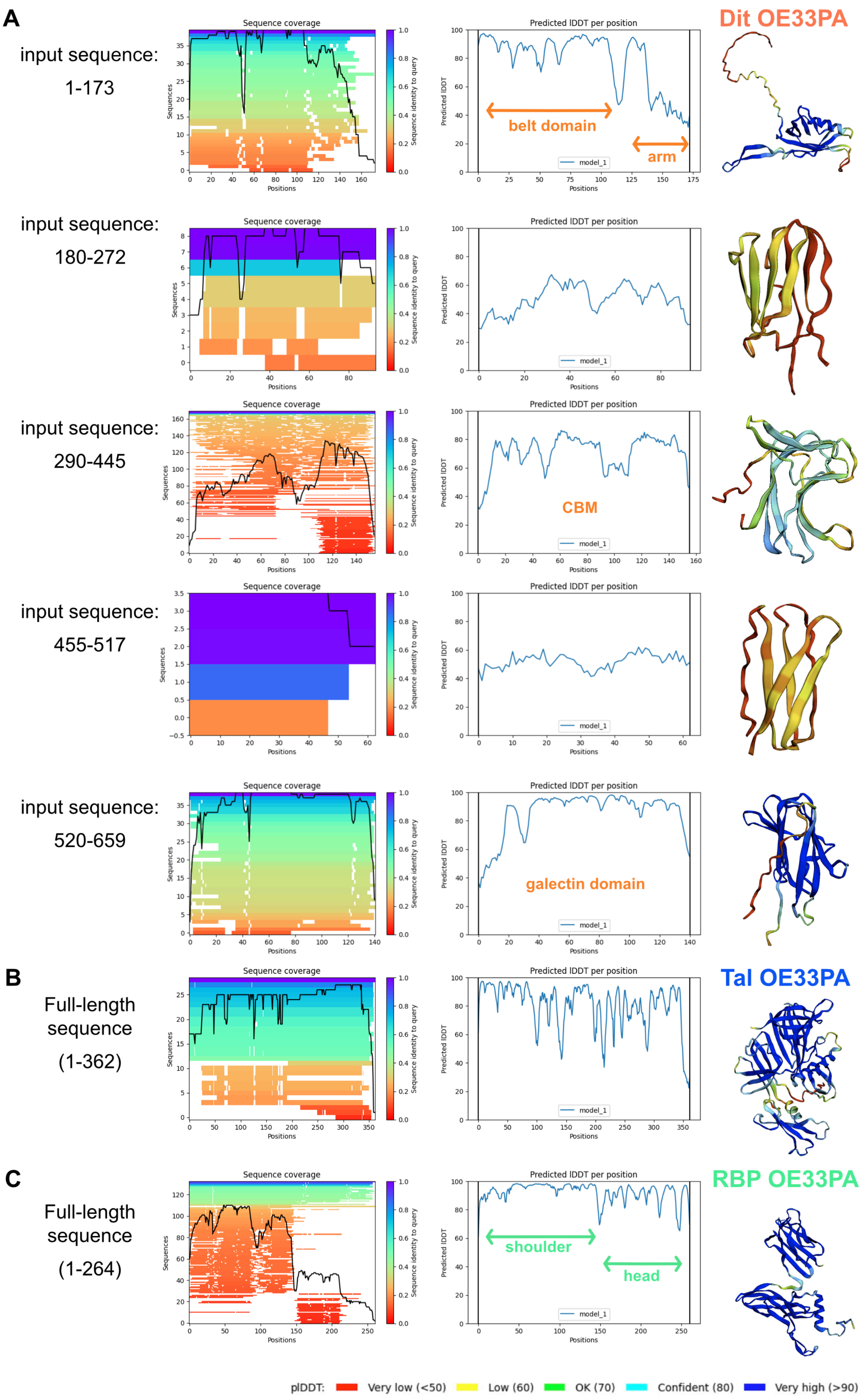

Figure S3. AlphaFold2 confidence measures for OE33PA Dit domains, Tal and RBP. of AlphaFold2 structure predictions of OE33PA Dit, Tal, and RBP. A. The Dit sequence was split into five parts and each of them were submitted to AlphaFold2. Left. The plots report the number of sequences, sequence coverage, and sequence identity to query. Right. Predicted

lDDT per position. AlphaFold2 predicted 3D structures are shown (the color code is indicated). B. The full-length Tal sequence was submitted to AlphaFold2. Left. This plot reports the number of sequences, sequence coverage, and sequence identity to query. Right. Predicted lDDT per position. The AlphaFold2 predicted 3D structure is shown (the color code is indicated). C. The full-length RBP sequence was submitted to AlphaFold2. Left. This plot reports the number of sequences, sequence coverage, and sequence identity to query. Right. Predicted plDDT per position. The AlphaFold2 predicted 3D structures is shown (the color code is indicated).

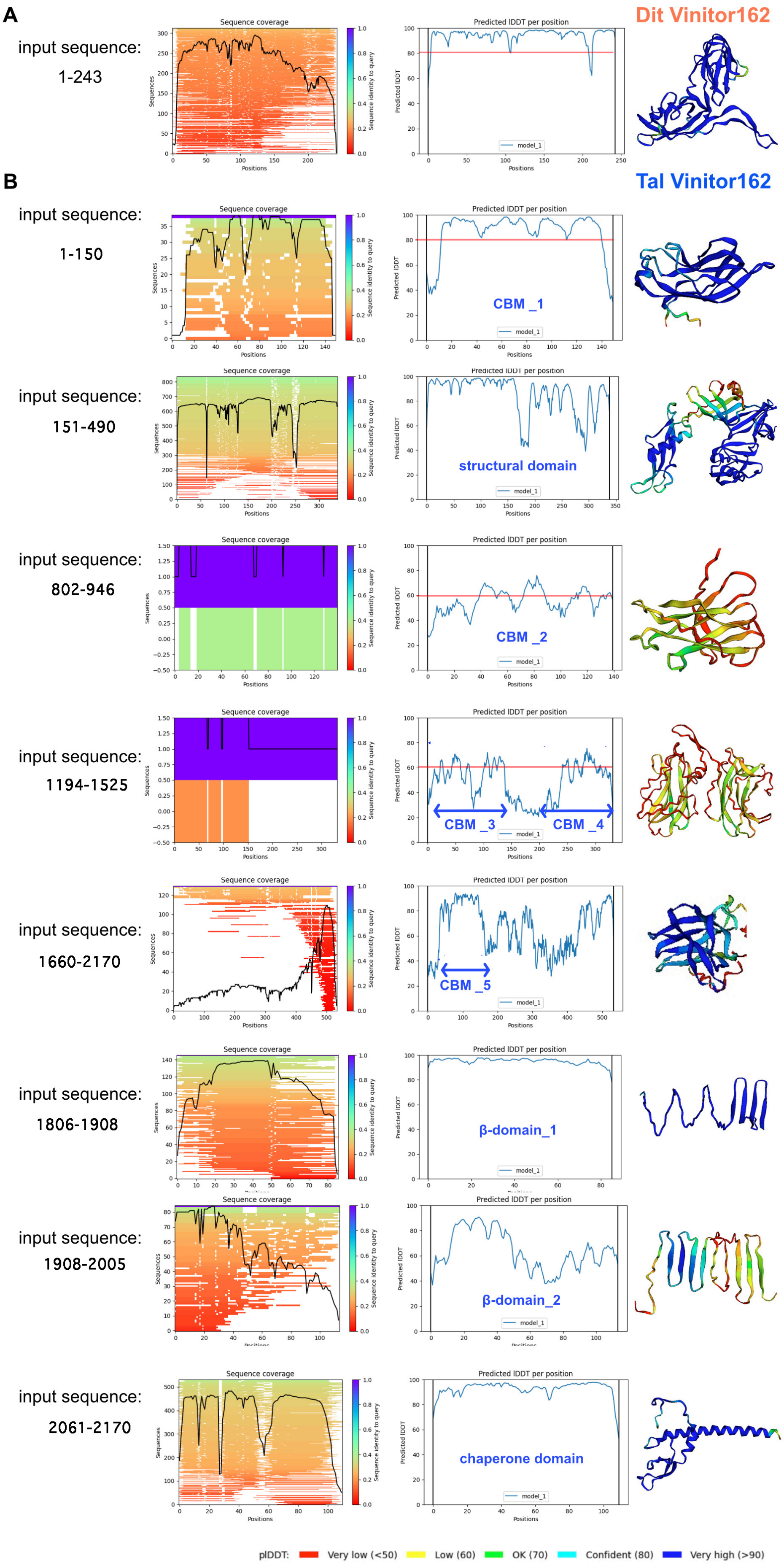

Figure S4. AlphaFold2 confidence measures for Vinitor162 Dit and Tal domains. A. The full-length Dit sequence was submitted to AlphaFold2. Left. This plot reports the number of sequences, sequence coverage, and sequence identity to query. Right. Predicted lDDT per

position. The AlphaFold2 predicted 3D structure is shown (the color code is indicated). B. The Tal sequence was split into eight parts and each of them were submitted to AlphaFold2. Left. The plots report the number of sequences, sequence coverage, and sequence identity to query. Right. Predicted lDDT per position. AlphaFold2 predicted 3D structures are shown (the color code is indicated).

Table S1. Comparison of domain predictions by HHpred and AlphaFold2.

Domains	HHpred boundaries (PDB ID; probability)	AlphaFold2 boundaries	Dali PDB ID•Z-score• rmsd (Å)•aligned residues
**OE33PA Dit**			
**belt**	1-166 (2wzp, *L. lactis* phage p2; 99.9%)<br>43-281 (2x8k, *B. subtilis* phage SPP1; 92.9%)	1-131	2wzp (*L. lactis* phage p2)• Z=13.4•rmsd=2.3•121<br>2x8k (*B. subtilis* phage SPP1)• Z=9.5•rmsd=3.4•112
**unknown β-fold**	n.i	199-270	2x5p (fibronectin-binding domain)• Z=6•rmsd=3•75
**CBM**	286-438 (2w5f, CBM22 CAZyDB; 95.1%)	305-445	5w6h (tail spike protein)• Z=12.2•rmsd=2.6•131<br>1gui (CBM4 CAZyDB)• Z=11.8•rmsd=2.3•130
**unknown β-fold**	n.i	455-517	nd
**galectin**	554-657 (2x8k, *B. subtilis* phage SPP1; 96.2%)	527-659	2x8k (*B. subtilis* phage SPP1)• •Z=12.3•rmsd=2.6•119<br>2wzp (*L. lactis* phage p2)• •Z=11.9•rmsd=2.6•121
**OE33PA Tal**			
**structural domain (phage T4 gp27-like)**	2-359 (3gs9, *Listeria* phage; 99.6%)<br>27-348 (3d37, *Neisseria* phage; 99.4%)<br>5-351 (6v8i, *Staphylococcus* phage 80α; 99.2%)<br>1-347 (1wru, phage Mu; 99.1%)<br>1-349 (3cdd, phage MuSo2; 99.1%)	1-362	2wzp (*L. lactis* phage p2)• Z=19.7•rmsd=4.4•300<br>3d37 (*Neisseria* phage)• Z=16.3•rmsd=4•267<br>3cdd (phage MuSo2)• Z=14.9•rmsd=4.1•246<br>3gs9 (*Listeria* phage)• Z=13.9•rmsd=3.8•263
**OE33PA RBP**			
**shoulder**	1-147 (4l9b, *L. lactis* phage 1358; 99.94%)	1-148	4l92 (*L. lactis* phage 1358)• Z=14.9•rmsd=2.2•144
**head**	155-261 (6r5w, *Listeria* phage PSA; 98.4%)<br>167-260 (4ios, *L. lactis* phage TP-901; 98.4%)<br>130-260	173-261	2fsd (*L. lactis* phage Bil170)• Z=11.9•rmsd=2.5•97<br>6r5w (*Listeria* phage PSA)• Z=10.7•rmsd=2.3•96

	(2fsd, *L. lactis* phage Bil170; 97.7%)		
	1-260<br>(1zru, *L. lactis* phage; 99.98%)		
**Vinitor162 Dit**			
**belt and galectin**	1-243<br>(2x8k, *B. subtilis* phage SPP1; 100%)	1-242	2x53 (*L. lactis* phage p2)•<br>Z=12.9•rmsd=3.2•210
**Vinitor162 Tal**			
**CBM_1**	n.i	1-150	1us2 (CBM15 CAZyDB)•<br>Z=8.3•rmsd=2.6•104
**structural domain (phage T4 gp27-like)**	129-489<br>(3gs9 *Listeria* phage; 99.9%)<br>(6v8i, *Staphylococcus* phage 80α; 99.7%)	151-489	3gs9 (*Listeria* phage)•<br>Z=18.3•rmsd=3.8•270
**α-helix_1**	490-708<br>(7boz, phage T7 tail fiber; 95.8%)	490-801	nd
**CBM_2**	827-940<br>(4xup, CBM22 CAZyDB; 69%)<br>(5w6h, coliphage CBA120 tail spike; 50.6%)	802-946	4csb (bacterial β-barrel)•<br>Z=4.1•rmsd=3•113
**unknown β-fold**	n.i	947-1193	nd
**CBM_3**	1194-1333<br>(4xup, CBM22 CAZyDB; 96.9%)<br>(6zpv, GH51 CAZyDB; 95.6%)	1194-1333	5w6h (coliphage CBA120 tail spike)•<br>Z=14.3•rmsd=2.3•126<br>2wze (CBM22 CAZyDB)•<br>Z=12.5•rmsd= 2.5•125
**linker_2**		1334-1407	
**CBM_4**	1407-1525<br>(4xup, CBM22 CAZyDB; 96.9%)	1408-1524	4bjo (CBM4 CAZyDB)•<br>Z=8.8 • rmsd=2.0•97
**unknown β-fold**	n.i	1525-1636	nd
**CBM_5**	1681-1784<br>(4d0q, CBM; 76.1%)<br>(1dyo, CBM22 CAZyDB; 49.2%)	1637-1791	1dyo (CBM22 CAZyDB)•<br>Z=9.4• rmsd=2.8•116
**β-domain_1**	n.i	1806-1891	4s37 (membrane-piercing spike)•<br>Z=5.1• rmsd=1.5•52
**β-domain_2**	n.i	1908-2005	4bxq•<br>Z=5.7• rmsd=3.7•86
**C-term**	2065-2170<br>(6f45, phage S16 tail fiber; 96.6%)<br>(3gw6, tail spike; 94.8%)	2061-2170	4uw8 (phage T5 tail fiber)•<br>Z=5.4• rmsd=3.1•81<br>3gw6 (tail spike)•<br>Z=5.8• rmsd=2.8•90
